# Shotgun proteomics coupled to nanoparticle-based biomarker enrichment reveals a novel panel of extracellular matrix proteins as candidate serum protein biomarkers for early-stage breast cancer detection

**DOI:** 10.1186/s13058-020-01373-9

**Published:** 2020-12-02

**Authors:** Claudia Fredolini, Khyatiben V. Pathak, Luisa Paris, Kristina M. Chapple, Kristine A. Tsantilas, Matthew Rosenow, Tony J. Tegeler, Krystine Garcia-Mansfield, Davide Tamburro, Weidong Zhou, Paul Russo, Samuele Massarut, Francesco Facchiano, Claudio Belluco, Ruggero De Maria, Enrico Garaci, Lance Liotta, Emanuel F. Petricoin, Patrick Pirrotte

**Affiliations:** 1 Center for Applied Proteomics & Molecular Medicine, George Mason University, Manassas, VA USA; 2 Collaborative Center for Translational Mass Spectrometry, Translational Genomics Research Institute, 445 N 5th St, Phoenix, AZ 85004 USA; 3 Department of Surgical Oncology, Centro di Riferimento Oncologico di Aviano (CRO) IRCCS, 33081 Aviano, PN Italy; 4 Dipartimento di Oncologia e Medicina Molecolare, Istituto Superiore di Sanità, Rome, Italy; 5 Istituto di Patologia Generale, Università Cattolica del Sacro Cuore, 00168 Rome, Italy; 6 Fondazione Policlinico Universitario “A. Gemelli” - I.R.C.C.S, 00168 Rome, Italy; 7 University San Raffaele and Istituto di Ricovero e Cura a Carattere Scientifico San Raffaele, Rome, Italy

**Keywords:** Invasive ductal carcinoma, Mammography, Serum, Protein enrichment, Nanoparticles, Multiple reaction monitoring

## Abstract

**Background:**

The lack of specificity and high degree of false positive and false negative rates when using mammographic screening for detecting early-stage breast cancer is a critical issue. Blood-based molecular assays that could be used in adjunct with mammography for increased specificity and sensitivity could have profound clinical impact. Our objective was to discover and independently verify a panel of candidate blood-based biomarkers that could identify the earliest stages of breast cancer and complement current mammographic screening approaches.

**Methods:**

We used affinity hydrogel nanoparticles coupled with LC-MS/MS analysis to enrich and analyze low-abundance proteins in serum samples from 20 patients with invasive ductal carcinoma (IDC) breast cancer and 20 female control individuals with positive mammograms and benign pathology at biopsy. We compared these results to those obtained from five cohorts of individuals diagnosed with cancer in organs other than breast (ovarian, lung, prostate, and colon cancer, as well as melanoma) to establish IDC-specific protein signatures. Twenty-four IDC candidate biomarkers were then verified by multiple reaction monitoring (LC-MRM) in an independent validation cohort of 60 serum samples specifically including earliest-stage breast cancer and benign controls (19 early-stage (T1a) IDC and 41 controls).

**Results:**

In our discovery set, 56 proteins were increased in the serum samples from IDC patients, and 32 of these proteins were specific to IDC. Verification of a subset of these proteins in an independent cohort of early-stage T1a breast cancer yielded a panel of 4 proteins, ITGA2B (integrin subunit alpha IIb), FLNA (Filamin A), RAP1A (Ras-associated protein-1A), and TLN-1 (Talin-1), which classified breast cancer patients with 100% sensitivity and 85% specificity (AUC of 0.93).

**Conclusions:**

Using a nanoparticle-based protein enrichment technology, we identified and verified a highly specific and sensitive protein signature indicative of early-stage breast cancer with no false positives when assessing benign and inflammatory controls. These markers have been previously reported in cell-ECM interaction and tumor microenvironment biology. Further studies with larger cohorts are needed to evaluate whether this biomarker panel improves the positive predictive value of mammography for breast cancer detection.

## Background

Invasive ductal carcinoma (IDC) is the most common type of breast cancer, accounting for 80% of all breast cancers and affecting women at any age. For asymptomatic individuals, annual or biennial mammographic screening is the primary tool for prevention and early diagnosis of breast cancers. However, several trials have called into question the efficacy of mammographic screening to reduce mortality. False positives and false negatives represent a clear limitation of mammography screening. The incidence of IDC, and its associated mortality, has not decreased in the past 10 years despite large-scale mammographic screening efforts [1]. Specifically, in current mammographic image-based screening approaches, lack of sensitivity to detect many early-stage breast cancer is concomitant with increased frequency of unnecessary biopsies (biopsies of benign lesions) [2], over-diagnosis, and over-treatment [1, 3, 4]. It has been proposed that the consequences of over-treatment, such as complications from surgery, cardiotoxicity, and cardiovascular disease have offset the expected reduction in mortality resulting from mammographic screening efforts [3].

Despite technological advances in screening mammography between 2005 and 2013 [5], breast cancer detection rates only marginally increased (31.5 to 34.7%). Surprisingly, an increase in abnormal interpretations was recorded during the same time span (from 8 to 12.6%), alongside a decrease in positive predictive values (PPV) of biopsy recommendations and performed biopsies (31.5% down to 27.5% and 39.5% down to 30.4%, respectively) [6]. Moreover, current screening approaches reportedly have a low negative predictive value (NPV), missing approximately 20% of aggressive subtypes of IDC [7]. In these subtypes specifically, high breast density, a well-known risk factor for aggressive breast cancer, can reduce the ability of screening methods to detect cancer and lead to false negative results [8].

The availability of a robust, minimally invasive, and clinically actionable blood-based test to support and confirm mammography breast cancer screening results would be of great utility to increase both PPV and NPV especially for early-stage IDC detection. In particular, identification of a robust blood-based molecular signature of breast cancer could complement imaging-based screening approaches and would likely reduce false positive and false negative results when used together. So far, however, no blood-based clinical assay based on a single protein or panel of proteins has demonstrated sufficient diagnostic specificity and sensitivity to detect early-stage breast cancer in a clinical setting [9, 10].

Our study objectives aimed at identifying blood-based low molecular weight (LMW) proteins and protein fragments that show the potential to be used in parallel to mammography screening as serum biomarkers for early-stage breast cancer. Our final goal was to perform discovery, qualification, and verification of a panel of breast cancer candidate biomarkers using two independent mass spectrometry method and independent sample sets.

Blood levels of early-stage cancer biomarkers are expected to be low. In this study, we employed hydrogel nanoparticles (HNs) as a cutting-edge sample preparation technology, which we have specifically engineered to capture and enrich low abundance and LMW proteins in biofluids such as blood [11–14]. Our biomarker discovery efforts focus on the LMW portion of the proteome because any cancer biomarker that originates in the affected organ must be able to effectively traverse the endothelial cell wall barrier of the vasculature that provides a size selection of biomarkers below the MW of albumin [11].

In order to demonstrate breast cancer specificity, in addition to our breast cancer cohort, our discovery efforts utilized blood samples taken from patient cohorts with other solid cancers, including colon, ovarian, prostate and lung cancer, and melanoma. Moreover, in order to analyze the performance of the selected candidate markers and minimize the evaluation of those that associate with inflammation and benign pathologies, the verification set included a series of benign and inflammatory controls. If further validated in the future, these markers could augment mammographic screening in a clinical setting.

## Materials and methods

### Materials

N-isopropylacrylamide (NIPAm), N,N′ methylene bisacrylamide (BIS), allylamine (AA), potassium persulfate (KPS), vinyl sulfonic acid (VSA), Cibacron Blue F3GA (CB), dithiothreitol (DTT), iodoacetamide (IAA), urea, tris-HCl, sodium thiocynate (NaSCN), tris-(2-carboxyethyl)phosphine (TCEP), and ammonium hydroxide were procured from Sigma-Aldrich (St. Louis, MO). All solvents (water, acetonitrile [ACN], formic acid) were LC-MS grade and obtained from Fisher Scientific (Waltham, MA). Red top glass vacutainer tubes for serum separation were purchased from Becton, Dickinson and Company (Franklin Lakes, NJ). Stromal cell-derived factor 1β (SDF-1β; MW 11 kDa) was from Antigenix America (Melville, NY), insulin-like growth factor 1 (IGF1; MW 22 kDa) was from AbD Serotec (Raleigh, NC), insulin-like growth factor-binding protein 7 (IGFBP7; MW 29 kDa) was from PreproTech (Rocky Hill, NJ), and chicken lysozyme was from Sigma-Aldrich. All synthetic peptides were procured from Thermo Fisher.

### Study cohorts and sample collection

#### Discovery set

**Fig. 1:**
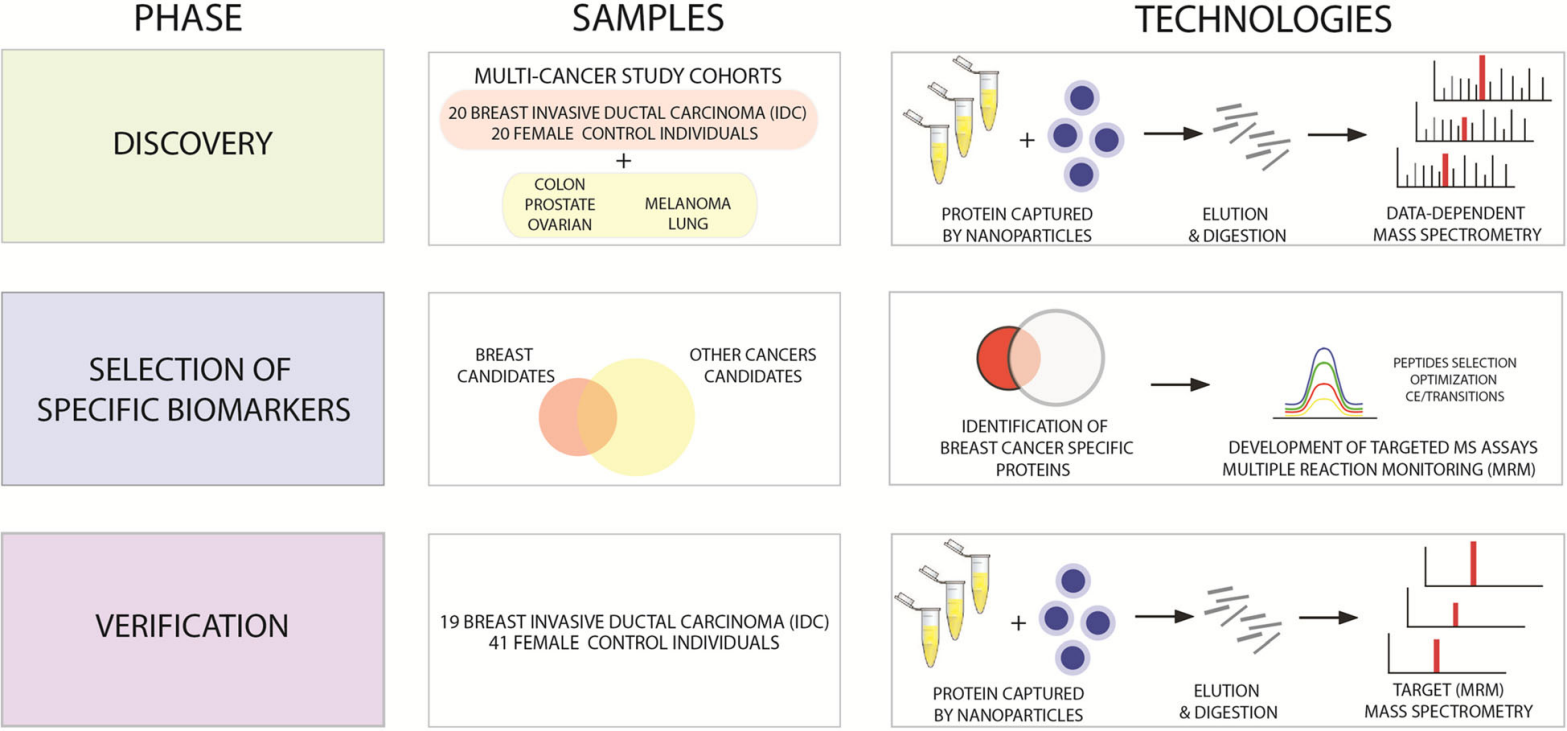
Workflow for biomarker discovery and verification based on nanoparticle enrichment and shotgun and targeted mass spectrometry

Our discovery study set used serum samples obtained from six cancer patient cohorts (breast, lung, colon, melanoma, prostate, and ovary) maintained by the Italian National Serum Bank hosted at Ospedale Maggiore Policlinico of Milan (Fig. 1). Each cohort included 20 cancer patients (cases) and 20 benign controls (total *N* = 240 samples). The controls were matched to each cancer cohort by median age and range, smoking habits, benign pathologies including those that could confound imaging results, and gender. The breast cancer cohort included female patients with IDC at stage I, II, or III, according to the TNM system. All patients in the cohort had suspicious or highly suspicious abnormalities at mammography (BI-RADS score 4–5). Moreover, all patients were treatment-naïve at the time of collection with regard to chemotherapy, radiation, and surgery. Whole blood (8 mL) was collected in red top glass vacutainer tubes, clotted for at least 30 min at room temperature, and centrifuged at 1500 rcf for 10 min. The serum samples were transferred to pre-labeled cryo-tubes and stored promptly at − 80 °C. The samples were shipped on dry ice to the Italian National Serum Bank. All procedures were performed in accordance with the ethical standards laid down in the 1964 Declaration of Helsinki and its later amendments or comparable ethical standards. All specimens and clinical data were collected under an IRB-approved protocol between 2005 and 2007. Characteristics of patients involved in the study are described in Table 1 (breast cancer cohort) and Supplementary Table S1 (other cancer cohorts).

**Table 1:** Characteristics of female breast cancer patients in the discovery and validation sets

Baseline variable	Discovery set		Validation set	
	Cases	Controls	Cases	Controls
	(***n*** = 20)	(***n*** = 20)	(***n*** = 19)	(***n*** = 41)
**Sex** (Female)	20	20	19	41
**Mean age (years)**	59.2 ± 11.4	60.1 ± 7.3	60 ± 15.4	51 ± 6.3
**Suspicious mammography with negative cytology**				**4**
**Smoking status**				
Never	12	19		19
Former	3			7
Current	4	1		11
NA	1			4
**Clinical stage**				
0 Tis N0 M0				
I T1 N0 M0	10		19	
IIA T1 N1 M0	5			
IIB T2 N1 M0	2			
IIIA T0 N2 M0	3			
**ER**				
Positive/negative/NA	9/−/11		10/4/5	
**PgR**				
Positive/negative/NA	8/−/12		9/5/5	
**Her2 Neu**				
Positive/negative/NA	10/7/3		10/4/5	
**Histologic grading**				
G1/G2/G3/NA	0/8/5/7		2/12/5/0	
**Molecular subtype**				
Luminal/Her2-enriched/triple-negative/NA			10/3/1/5	
**Comorbidities**				
None reported		17		22
Diabetes (type I or type II)				3
Allergy				1
Thyroid nodules				1
Hypothyroidism		1		3
Chronic gastritis				1
Depressive disorder				2
Hypertension		1		4
Hypotension				1
Osteoporosis				1
Hypocholesterolemia		1		
Epilepsy		1		
Arthrosis				2
Asthma				1

#### Verification set

The verification cohort (*N* = 60) included blood samples taken only from patients diagnosed with IDC at stage I (T1N0M0) along with samples from control individuals that included women who had positive mammograms with benign pathology at biopsy and women with minor pathologies such as hypertension and high cholesterol (Table 1). We applied the following inclusion criteria to determine study eligibility: available mammogram data (suspicious or highly suspicious abnormalities at mammography, BI-RADS score 4–5), age 33–89 years, and available outcome data.

### HN synthesis

HNs functionalized with Cibacron Blue (CB) and coated with a vinylsulfonic acid (VSA) containing shell were synthetized as previously described by Tamburro et al. [11]. Briefly, N-isopropylacrylamide (NIPAm)-allylamine (AA) particles were synthesized by precipitation polymerization and then covalently functionalized with CB by nucleophilic substitution. NIPAm (Sigma-Aldrich (0.89 g, 7.83 mmol, 7.83 mmol)) and N,N′ methylene bisacrylamide (BIS; Sigma-Aldrich (0.042 g, 0.27 mmol)) were dissolved in water, and the solution was filtered and purged with nitrogen at room temperature. AA was then added (0.051 g, 0.90 mmol), followed by potassium persulfate (KPS) (0.0070 g, 0.025 mmol) to activate polymerization. The reaction was maintained at 75 °C under nitrogen for 3 h. Poly (NIPAm-co-AA) particles were washed by centrifugation to eliminate unreacted monomer. CB dissolved in sodium carbonate was added (0.76 g, 0.90 mmol) to the suspension and stirred at room temperature under nitrogen for 48 h. The resulting poly (NIPAm/CB) particles were first washed with water, and then exposed to a new polymerization reaction. A solution containing 20 ml of poly (NIPAm/CB) particles, NIPAm (0.156 g, 1.38 mmol), BIS (0.013 g, 0.084 mmol), and VSA (26 μl, 0.344 mmol) was purged with nitrogen and then heated to 70 °C, and polymerization was induced by adding KPS (0.092 g, 0.328 mmol). The reaction was performed at 70 °C under nitrogen for 6 h. Newly synthetized poly (NIPAm/CB) core–poly(NIPAm-co-VSA) shell particles were washed in water by centrifugation (16,100 rcf, 50 min, 25 °C).

### HN-mediated enrichment of low-abundance plasma serum proteins

HNs offer several advantages over other methods used to enrich low abundant serum proteins: (i) Low abundance protein capture and enrichment is not based on immunodepletion methods but on the use of high affinity dye based selective capture in the core of the HN particle with simultaneous size-based exclusion of abundant proteins that often act as a carrier for low abundant proteins that may be lost during the immunodepletion process; (ii) HNs have been proven to protect proteins from enzymatic degradation during sample preprocessing [15]; (iii) HNs has shown to perform better in enriching low molecular weight (LMW; MW ≤ 40 kDa) when compared to methods based on peptide enrichment, or ultrafiltration [16].

Serum samples from the six discovery cohorts were processed by incubation with HNs in a randomized order. The serum samples (0.5 mL) were thawed, centrifuged (7 min, 4 °C, 16,100 rcf), and diluted with 1 mL 50 mM Tris-HCl, pH 7.0. SDF-1β, IGF1, and IGFBP7 were spiked in each sample as internal standards for quality and process control.

In the discovery set, both case and control samples contained IGF1 at 2 μg/μL, whereas IGFBP7 was spiked at 2 μg/μL in the case samples and 0.2 μg/μL in the control samples, and SDF-1β was spiked at 40 ng/μL in the control samples and 4 ng/μL in the case samples. Each sample was then incubated with 0.5 mL HNs (HN-CB/VSA, 7 mg/mL dry weight) for 30 min at room temperature. The samples were centrifuged (16,100 rcf, 25 °C, 20 min) and washed with 0.25 M NaSCN followed by two washes with water. The proteins were eluted by incubating the particles for 15 min at room temperature with 600 μL elution buffer (70% acetonitrile, 10% NH_4_OH) followed by centrifugation (16,100 rcf, 25 °C, 10 min). The elution step was repeated twice, and the eluates were combined, dried in a vacuum concentrator, and stored at − 20 °C until use.

For verification, chicken lysozyme was spiked at 0.2 μg/mL and was used as internal standard. Three hundred microliters of serum was diluted with 600 μL of 50 mM Tris-HCl, pH 7.0, and incubated with 300 μL of HN-CB/VSA particles (15 min, 25 °C) on a shaker. The nanoparticles were centrifuged at 16,100 rcf (30 min, 25 °C) and rinsed twice with 500 μL of 50 mM Tris-HCl, pH 7.0. The supernatant was discarded and the pelleted particles were incubated in 500 μL elution buffer (500 mM NaCl, 5 mM EDTA, 2% sodium deoxycholate in 50 mM Tris, pH 8.8) on a rocking shaker (47 rcf, 30 min, 60 °C). The samples were spun down (16,100 rcf, 15 min, 25 °C), and the supernatants were collected and vacuum-dried.

### Protein digestion and LC-MS/MS analysis

#### Discovery set

All 240 samples analyzed from the discovery set were analyzed by LC-MS/MS in a randomized order to minimize batch effect and run bias. Dried protein eluates were reconstituted in 8 M urea, reduced in 10 mM dithiothreitol (DTT, Sigma), alkylated in 50 mM iodoacetamide (IAA, Sigma), and digested overnight in trypsin using an enzyme-to-protein ratio of 1:25 (v/v) (Promega Corporation, Madison, WI) at 37 °C. Tryptic peptides were further purified by C18 ZipTips (Millipore, Burlington, MA) and separated on a C18 analytical column (0.2 × 50 mm, Michrom Bioresources, Inc., Auburn, CA) using a HPLC Surveyor MS pump plus and Micro AS autosampler (Thermo Fisher) coupled to an LTQ-Orbitrap mass spectrometer (Thermo Fisher Scientific, San Jose, CA). After sample injection, the column was washed for 2 min with mobile phase A (0.1% formic acid) and the peptides were eluted using a linear gradient of 0% mobile phase B (0.1% formic acid, 80% acetonitrile) to 50% mobile phase B in 90 min at 500 nL/min, and then 100% mobile phase B for an additional 5 min. The mass spectrometer was operated in data-dependent mode where the top five most abundant molecular ions were dynamically selected for collision-induced dissociation (CID) using a normalized collision energy (CE) of 35%. Raw files were searched against the NCBI human database with SEQUEST (Thermo Fisher Scientific). The following settings were applied: two missing cleavages allowed, methionine oxidation as a variable modification and cysteine carbamidomethylation as a fixed modification. High-confidence peptide identifications were obtained by applying the following filter criteria to the search results: Xcorr versus charge ≥ 1.9, 2.2, 3.5 for 1+, 2+, 3+ ions; ΔCn >  0.1; probability of randomized identification ≤ 0.01. Downstream differential analysis was based on spectral counts, as computed by Scaffold (Proteome Software, Inc., Portland, OR). The scaffold settings were protein and peptide probability threshold of 95%, 1 peptide per protein, and 1% false discovery rate.

#### Verification set

Protein eluates were quantified for total protein yield and normalization using reverse phase protein array analysis as previously described [17], and then reduced (5 mM TCEP, 40 min, 60 °C) and alkylated (10 mM IAA, 37 °C) in the dark. The samples were then diluted 4-fold in 50 mM Tris-HCl (pH 7.0), and 250 ng/μL trypsin Gold (Promega) was added at an enzyme-to-protein ratio of 1:50 (*v/v*). After 3 h of incubation at 37 °C, fresh trypsin was added to a final enzyme-to-protein ratio of 1:25 (*v/v*) and incubation was continued overnight (8 h, 63 rcf, 37 °C). Heavy-labeled peptides, GYSLGNWVCAA[^13^C_6_^15^N_2_(K)] and FESNFNTQATN[^13^C_6_^15^N_4_(R)], corresponding to GYSLGNWVCAAK and FESNFNTQATNR (Supplementary Table S2) for chicken LYZ were spiked in to a final concentration of 1750 fmoles. The samples were acidified with 50% formic acid to a final concentration of 1%, sieved through an AcroPrep 96-well plate (Pall Corporation, Port Washington, NY) to remove precipitated deoxycholate, and desalted using 1 cc Sep-Pak C18 (Waters, Milford, MA) solid-phase extraction columns. Finally, the eluted peptides were vacuum-dried and stored at − 20 °C until LC-MRM analysis.

The dried peptides were reconstituted in 0.1% formic acid and analyzed using online liquid chromatography on a nanoACQUITY UPLC (Waters) coupled to a Xevo TQ-s triple quadrupole mass spectrometer (Waters) with nano ESI in positive mode. Chromatography separation was performed on a BEH C18, 1.7 μm, 0.1 × 100 mm analytical column (Waters) using an 83.5 min gradient from 3 to 90% mobile phase B (acetonitrile, 0.1% formic acid) and 97 to 10% mobile phase A (water, 0.1% formic acid) at a flow rate 0.5 μL/min. The following gradient conditions were used: 3 to 7% mobile phase B for 1 min, 7 to 25% mobile phase B for 1 to 72 min, 25 to 45% mobile phase B for 10 min, 45 to 90% mobile phase B for 0.5 min, 90% B for 0.5 min, and column equilibration on 3% B for 10 min. Mobile phase A blanks were injected alternatively after every sample to prevent any carryover, column clogging, and rise in column pressure during the data acquisition. The *E. coli* tryptic digests (250 ng, Waters) bracketed every five patient sample injections (Supplementary Table S3) to assess instrumental variance. The measured variance remained under 15% coefficient of variation (CV) for the area under the curve throughout the acquisition. Peaks were integrated, and area-under-the-curve (AUC) values were calculated using the Skyline 3.5.0.9319 software package [20].

### LC-MRM optimization

Candidate peptides selected for LC-MRM verification are listed in Table 3. They were selected based on experimental (highest number of peptide identifications and intensities, with at least 3 highly abundant − *y* and − *b* ions) and computational (highest ESP scores [18], highest scored transitions using PeptideAtlas [19]) criteria. Although most peptides presented unique sequences, our panel contains several candidates with sequences shared by more than one protein or shared by their isoforms (Table 3). For each candidate marker, if available, several light and heavy peptides were synthesized following common empirical rules, such as size tolerance (7–15 amino acids) and absence of methionine of proline residues (Table 3, Supplementary Table S4). The LC-MRM assay was created using Skyline 3.5.0.9319 [20]. MRM transitions were optimized for charge state, cone voltage, and collision energy by direct infusion of synthetic light peptides at 5 μL/min, five-at-a-time at 200 nmoles in 10% acetonitrile, 0.1% formic acid, and a ramping collision energy from to 5 to 53 V. The most abundant precursor charge state within the mass range of *m/z* 400–1200 and *− y* type product ions were screened for each peptide. Of those, the three most abundant ions for each peptide with optimal cone voltage and collision energies were chosen (Supplementary Table S4). An equimolar mixture of all the synthetic peptides at 500 fmoles/peptide was subjected to unscheduled LC-MRM analysis (20 ms dwell time/transition) to identify optimal retention times with a window of ± 7.5 min. Heavy peptides corresponding to chicken LYZ were used for retention time alignment, determination of instrumental variance, and relative quantitation. Of these, the chicken LYZ peptide FESNFNTQATNR showed the least variation (CV = 18.07%) and was thus selected for normalization. Based on a known spike-in concentration and peak area ratio of light-to-heavy forms of the LYZ peptide, a normalization factor (concentration ratio of light-to-heavy/peak area ratio of light-to-heavy) was computed. This factor was applied for peptide normalization, and the normalized area for each candidate peptide was used for downstream data analysis.

### Data analysis

#### Discovery set

For each discovery set (breast cancer and other solid tumors), the relative difference (Diff [%]) between spectral counts in cases and controls was determined as follows:

$$ \mathrm{Diff}\ \left(\%\right)=\left[\left({\sum}_{i=1}^{i=n}\mathrm{spectral}\ \mathrm{counts}\ p\right)-\left({\sum}_{j=1}^{j=z}\mathrm{spectral}\ \mathrm{counts}\ p\right)\right]/\left[\left({\sum}_{i=1}^{i=n}\mathrm{spectral}\ \mathrm{counts}\ p\right)+\left({\sum}_{j=1}^{j=z}\mathrm{spectral}\ \mathrm{counts}\ p\right)\right]\frac{1}{2}\ast 100 $$

where

$$ {\displaystyle \begin{array}{c}\mathrm{spectral}\ \mathrm{counts}\ p=\mathrm{spectral}\ \mathrm{counts}\ \mathrm{corresponding}\ \mathrm{to}\ \mathrm{protein}\ p\\ {}n=\mathrm{number}\ \mathrm{of}\ \mathrm{cases}\\ {}z=\mathrm{number}\ \mathrm{of}\ \mathrm{controls}.\end{array}} $$

Proteins with Diff (%) ≥ 50% that were detected (≥ 1 spectral count) in at least 21% of cases or controls were considered candidate biomarkers. Peptides attributed to each biomarker were manually inspected to confirm raw spectral matching by SEQUEST. Candidate biomarkers found to be differentially abundant in other cancer cohorts were considered non-specific to breast cancer and removed from the candidate list. Internal spike-in process controls were monitored to assess case: control stoichiometry. Functional enrichment analysis comparing proteins identified in control or stage I, II, or III breast cancer samples was performed using Ingenuity Pathway Analysis (IPA) (QIAGEN, Redwood City, CA) using default IPA settings. Enriched pathways were filtered for significance (*p* < 0.05) and presence in > 50% of samples.

#### Verification set

We did not observe any batch effect in the verification dataset. The data obtained by MRM in the verification phase of the project were used to develop a statistical model to predict breast cancer. An initial statistical model (model 1) included all 41 candidate peptides corresponding to the 25 proteins of interest (Table 3). The probability for model entry was 0.05. A second logistic regression model (model 2) was computed with significant predictors from the first model entered simultaneously to obtain bootstrapped standard errors using 1000 samples: model sensitivity, specificity, PPV, NPV, and AUC. Data were analyzed using SPSS and STATA. Peptide significance (*p* < 0.05) was calculated by a Wilcoxon rank sum test and corrected for multiple testing using the Benjamini-Hochberg method (Supplementary Excel Table). Peptides were modeled in raw format and not transformed or dichotomized. Logistic regression models are reported using the observed coefficient and significance values with bootstrapped standard errors and confidence intervals.

## Results

### Discovery of IDC candidate biomarkers

To define differences in levels of circulating proteins predictive of organ-specific tumor growth, we analyzed six sets of serum samples from cancer patients (*n* = 20) and matched healthy controls (*n* = 20) using shotgun mass spectrometry. Proteins identified in IDC breast cancer patients (Table 1, Discovery set) were compared to proteins identified in patients affected by cancers of the colon, ovaries, lung, and prostate, as well as melanoma (Supplementary Table S1, Fig. 1).

For each organ-specific cancer cohort, we first evaluated relative differences between cases and controls for three spiked-in recombinant proteins—SDF-1β, IGF1, and IGFBP7, each of which served as internal quality control and was spiked in at a ratio case/control respectively of 1:10, 1:1, and 10:1. In the breast cancer cohort, the Diff (%) was − 124% for SDF-1β, 52% for IGF1, and 132% for IGFBP7, respectively, confirming that the approach can detect a 10-fold difference in protein levels, described by Diff (%) > 120%. Candidate biomarkers were selected in each discovery set based on a low stringency cut-off of 50%.

Fifty-six proteins showed a Diff (%) ≥ 50% between the IDC cases and matched benign controls (Table 2). Those proteins span a range of molecular weights from 10 to 532 kDa (median = 50 kDa). Proteins with a molecular weight between 20 and 60 kDa were preponderant (Supplementary Fig. 1A). The average absolute concentration in blood has not been reported for most of the 56 candidate markers. Therefore, we refer to their estimated abundance in parts per million, as reported by the PaxDB database (*H. sapiens* - Plasma (Integrated)) [21].Most have abundances in plasma below 5 ppm (Supplementary Fig. 1B).

**Table 2:** Breast cancer candidate serum biomarkers

#	Accession number	Uniprot ID	Gene	Description	MW (kDa)	Abundance (ppm)	GO-CC	Diff (%)	Cases (%)	Breast-specific	Novel
1	124248516	P59665	DEFA1	Alpha-defensin 1	10	138		63	53		
2	4759070	Q16627	CCL14	Chemokine (C-C motif) ligand 14 isoform 1 precursor	11	83	ES	164	32		
3	4507065	P03973	SLPI	Secretory leukocyte peptidase inhibitor precursor	14	9.52	EM	50	21	SPECIFIC	
4	4826898	P07737	PFN1	Profilin 1	15	207	EE	108	63	SPECIFIC	
5	5031635	P23528	CFL1	Cofilin 1 (non-muscle)	19	115	EM	133	58		
6	33946278	Q9Y281	CFL2	Cofilin 2	19	21.3	ES	80	37		
7	34850061	P62834	**RAP1A**	RAP1A, member of RAS oncogene family*	21	0.35	M	100	26	SPECIFIC	
8	4885375	P16403	HIST1H1C	Histone cluster 1, H1c	21	6.45	N	59	21	SPECIFIC	
9	4506413	P61224	RAP1B	RAP1B, member of RAS oncogene family-like	21	0.67	M	167	74	SPECIFIC	
10	148227764	Q93045	STMN2	Superiorcervical ganglia, neural specific 10	21	1.59	EE	120	53	SPECIFIC	
11	33695095	P13224	GP1BB	Glycoprotein Ib, beta polypeptide precursor	22	0.13	EE	133	21		
12	4504073	P61026	RAB10	Ras-related GTP-binding protein RAB10	22	0.11	M	145	58	SPECIFIC	
13	4507513	P35625	TIMP3	Tissue inhibitor of metalloproteinase 3 precursor	24	N/A	EM	53	47		NOVEL
14	4507651	P67936	TPM4	Tropomyosin 4 isoform 2	29	125	M	173	21	SPECIFIC	
15	24234708	Q99697	PITX2	Paired-like homeodomain transcription factor 2 isoform b	35	N/A	N	50	26		NOVEL
16	37550464	A6NMN3	FAM170B	PREDICTED:family with sequence similarity 170,member B	36	3.16	M	67	21		
17	209862875	Q7Z4I7	LIMS2	LIM and senescent cell antigen-like domains 2 isoform 1	38	0.43	M	200	21	SPECIFIC	
18	156523970	P02765	AHSG	Alpha-2-HS-glycoprotein	39	8613	ES	50	63	SPECIFIC	
19	156616273	P08567	PLEK	Pleckstrin	40	49.4	ES	143	32		
20	4501889	P63267	ACTG2	Actin, gamma 2 propeptide	42	25.6	ES	114	100		
21	20127528	P63261	ACTG1	Actin, gamma 1 propeptide	42	0.78	M	120	32		
22	4501887	Q9HBI1	PARVB	Parvin, beta isoform b	42	147	EM	112	100	SPECIFIC	
23	39725934	P36955	SERPINF1	Serine (or cysteine) proteinase inhibitor, clade F	46	3589	EM	111	21		
24	9966913	Q9P1U1	ACTR3B	Actin-related protein 3-beta isoform 1	48	0.08	EE	200	26	SPECIFIC	
25	55770868	I0CMK4	TUBB4Q	Tubulin, beta polypeptide 4, member Q	48	0.32		164	26		
26	17921989	Q6PEY2	TUBA3E	Tubulin, alpha 3e	50	3.5	EE	94	47	SPECIFIC	
27	46409270	Q9H4B7	TUBB1	Beta tubulin 1, class VI	50	1.63	N	98	63	SPECIFIC	
28	4507729	Q9BQE3	TUBA1C	Tubulin alpha 6	50	2.03	EE	57	21	SPECIFIC	
29	14210536	P68366	TUBA4A	Tubulin, alpha 4a	50	0.53	N	89	37	SPECIFIC	
30	14389309	Q13885	TUBB2A	Tubulin, beta 2	50	2.33	Mi	92	89	SPECIFIC	
31	13562114	Q9BUF5	TUBB6	Tubulin, beta 6	50	2.56	EE	72	68	SPECIFIC	
32	4503649	P00740	F9	Coagulation factor IX preproprotein	52	685	ES	200	21		
33	32483410	P38435	GC	Vitamin D-binding protein precursor	53	4435	M	200	26	SPECIFIC	
34	21071030	Q9Y243	AKT3	AKT3 kinase isoform 2	54	8804	ES	150	32	SPECIFIC	
35	32307163	P04217	A1BG	Alpha 1B-glycoprotein precursor	54	N/A	M	57	42		
36	148746204	Q9Y251	HPSE	Heparanase	61	0.02	ER	156	63		
37	13540563	Q9BXR6	CFHR5	Complement factor H-related 5	64	62.4	ER	67	26		
38	4504383	Q04756	HGFAC	HGF activator preproprotein	71	534	ES	76	79		
39	41281905	Q86UX7	FERMT3	Fermitin family homolog 3 long form	76	4.34	ER	123	89	SPECIFIC	
40	54607120	P02788	LTF	Lactotransferrin precursor	78	45.4	ES	51	63		
41	205277383	P26927	MST1	Macrophage stimulating 1 (hepatocyte growth factor-like)	82	269	ES	111	21	SPECIFIC	
42	119395709	P00488	F13A1	Coagulation factor XIII A1 subunit precursor	83	37.7	ER	63	21	SPECIFIC	
43	4504165	P06396	GSN	Gelsolin isoform a precursor	86	8905	ES	120	21	SPECIFIC	
44	47078292	P05106	ITGB3	Integrin beta chain, beta 3 precursor	87	1.46	EE	160	63	SPECIFIC	
45	4501891	P12814	ACTN1	Actinin, alpha 1 isoform b	103	6.5	ES	160	58		
46	5453579	P13497	BMP1	Bone morphogenetic protein 1 isoform 3 precursor	111	1.38	EM	133	26	SPECIFIC	
47	88758615	P08514	**ITGA2B**	Integrin alpha 2b preproprotein *	113	3.1	M	144	100	SPECIFIC	
48	7669550	P18206	VCL	Vinculin isoform meta-VCL	124	94.5	M	153	21		
49	40317626	P07996	THBS1	Thrombospondin 1 precursor	129	41.4	EM	94	100	SPECIFIC	
50	12667788	P35579	MYH9	Myosin, heavy polypeptide 9, non-muscle	227	2.67	EM	200	21	SPECIFIC	
51	223029410	Q9Y490	**TLN1**	Talin 1 *	270	22.5	ER	131	89	SPECIFIC	
52	156938343	Q9Y4G6	TLN2	Talin 2	272	0.32	M	114	42	SPECIFIC	
53	105990514	O75369	FLNB	Filamin B, beta (actin binding protein 278)	278	0.24	EM	86	32		
54	116063573	P21333	**FLNA**	Filamin A, alpha isoform 1 *	280	9.63	EM	154	89	SPECIFIC	
55	15147337	O95071	UBR5	Ubiquitin protein ligase E3 component n-recognin 5	309	0.03	M	59	42		
56	33350932	Q14204	DYNC1H1	Cytoplasmic dynein 1 heavy chain 1	532	0.04	EM	67	21	SPECIFIC	

Abundance (ppm): protein abundance in plasma according to the PaxDB integrated plasma database. *GO-CC* Gene Ontology category cellular component, *ES* extracellular space, *ER* extracellular region, *EM* extracellular matrix, *EE* extracellular exosomes, *M* membrane, *N* nucleus, *C* cytoskeleton, *Diff (%)* relative difference in abundance (percentage) between cases and controls, *Cases (%)* percentage of breast cancer cases in which the protein is present, *Breast-specific* increased abundance in breast cancer patients sera only, *Novel* not yet reported in PaxDB (plasma, mass spectrometry)

**Fig. 2:**
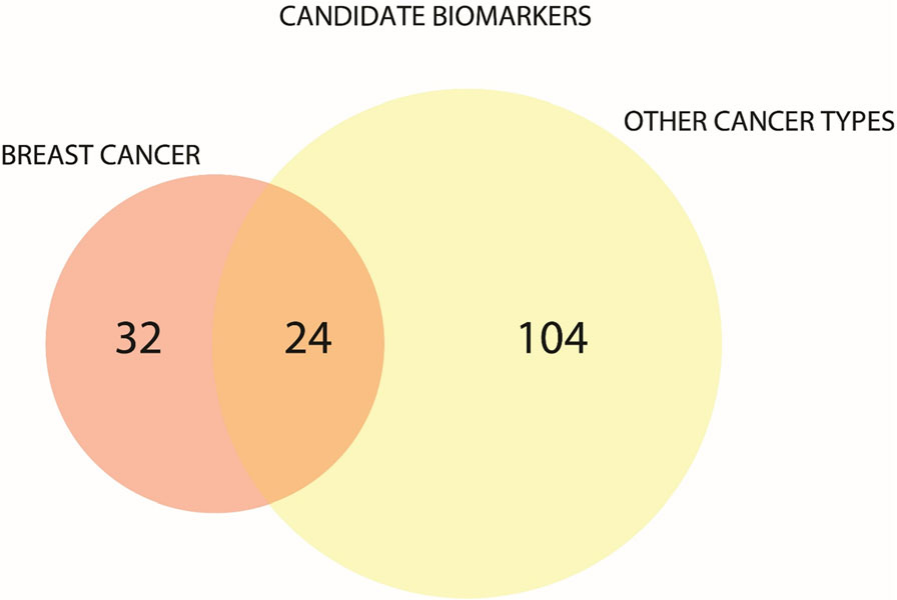
Venn diagram representing the number of specific and common candidate biomarkers identified in the study cohorts

Table 2 contains annotations for each of the 56 proteins. According to the gene ontology (GO) annotation, 37 (65%) of the 56 candidates are extracellular proteins and 14 (18%) are membrane proteins. Half of the annotations refer to roles in cell motility, such as cell-to-cell junctions, focal adhesions, and reorganization of the actin cytoskeleton (Supplementary Excel Table, “Gene ontology (GO)”). Seventeen of 56 (~ 30%) are known secreted proteins (Supplementary Excel Table “Subcellular location [CC]”). Two proteins, AKT3 and PITX2, were not previously reported in blood using mass spectrometry. Overall, 160 candidate serum biomarkers were identified across all 6 cohorts. Of those, 33 proteins were differentially abundant in IDC only (Fig. 2, Table 2).

Functional enrichment analysis for proteins identified in each disease subgroup in the breast cancer cohort (stages I, II, III, and control; Table 1, Discovery Set) yielded 38, 46, 36, and 49 pathways respectively, with significant overlap between groups (Supplementary Fig. 2). Enrichment of *Breast Cancer Regulation by Stathmin1* in breast cancer stages I and II was partly driven by 6 of the tubulin biomarker candidates. *Estrogen-Dependent Breast Cancer Signaling* was enriched in the control and stage III. *The Role of Tissue Factor in Cancer* was enriched in > 50% of samples only at stage I. Four of the seven proteins driving enrichment of *The Role of Tissue Factor in Cancer* are also candidate biomarkers: RAC-gamma serine/threonine-protein kinase (AKT3), Cofilin1 and 2 (CFL1, CFL2), and integrin beta 3 (ITGB3).

### Independent verification of candidate biomarkers and diagnostic models

**Fig. 3:**
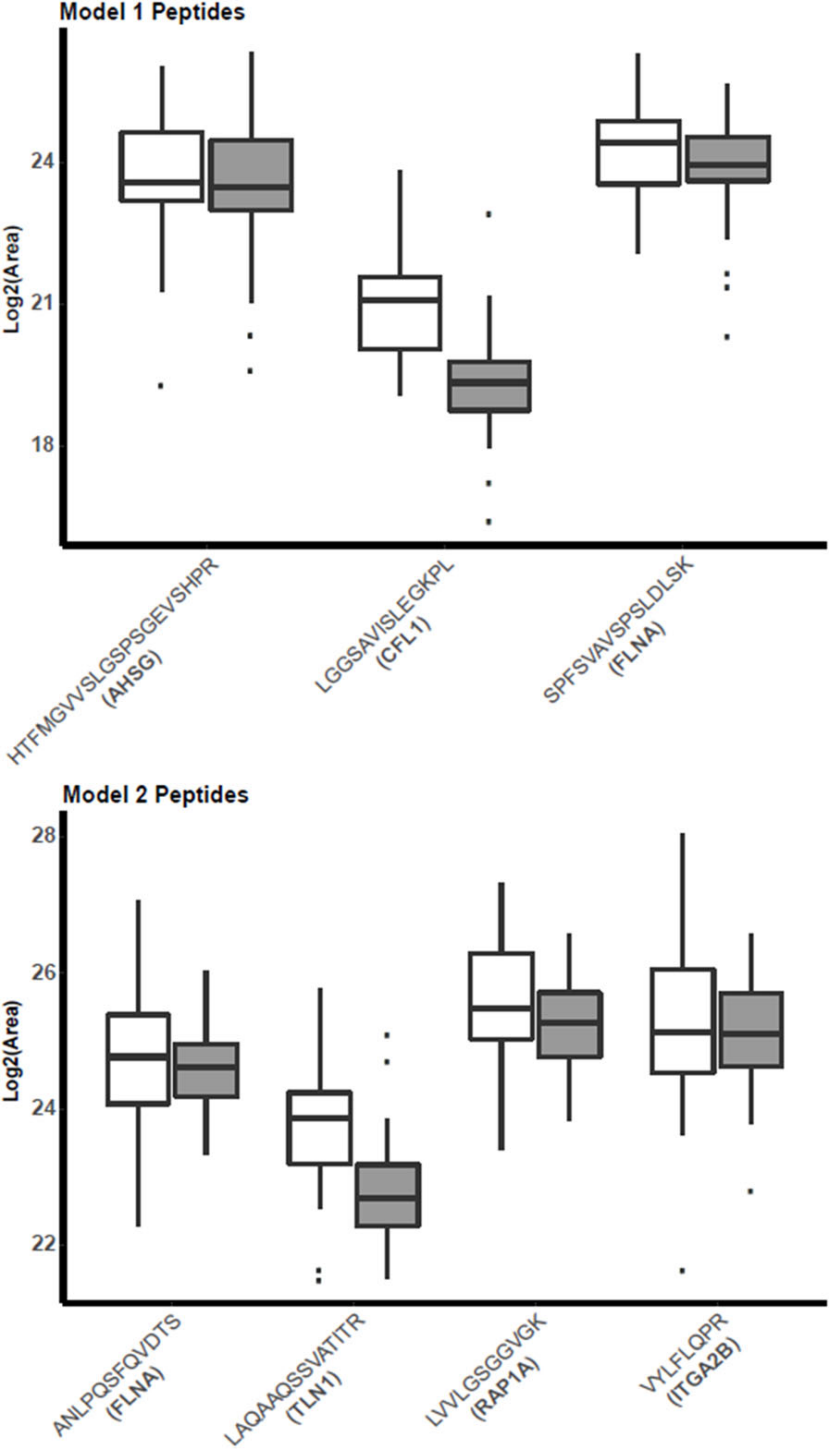
Serum protein levels measured by LC-MRM in the validation set. Normalized AUC values are shown for proteins in models 1 and 2 (cases = 19, white; controls = 41, gray). Adjusted *p* values were obtained using the Benjamini-Hochberg correction. ANLPQSFQVDTSK (FLNA, adjusted *p* value = 0.67); HTFMGVVSLGSPSGEVSHPR (AHSG, adjusted *p* value = 0.84); LAQAAQSSVATITR (TLN1, adjusted *p* value = 0.0019); LGGSAVISLEGKPL (CFL1, adjusted *p* value = 8.29E−5); LVVLGSGGVGK (RAP1A, *p* value = 0.34); SPFSVAVSPSLDLSK (FLNA, *p* value = 0.58) and VYLFLQPR (ITGA2B, *p* value = 0.69)

**Fig. 4:**
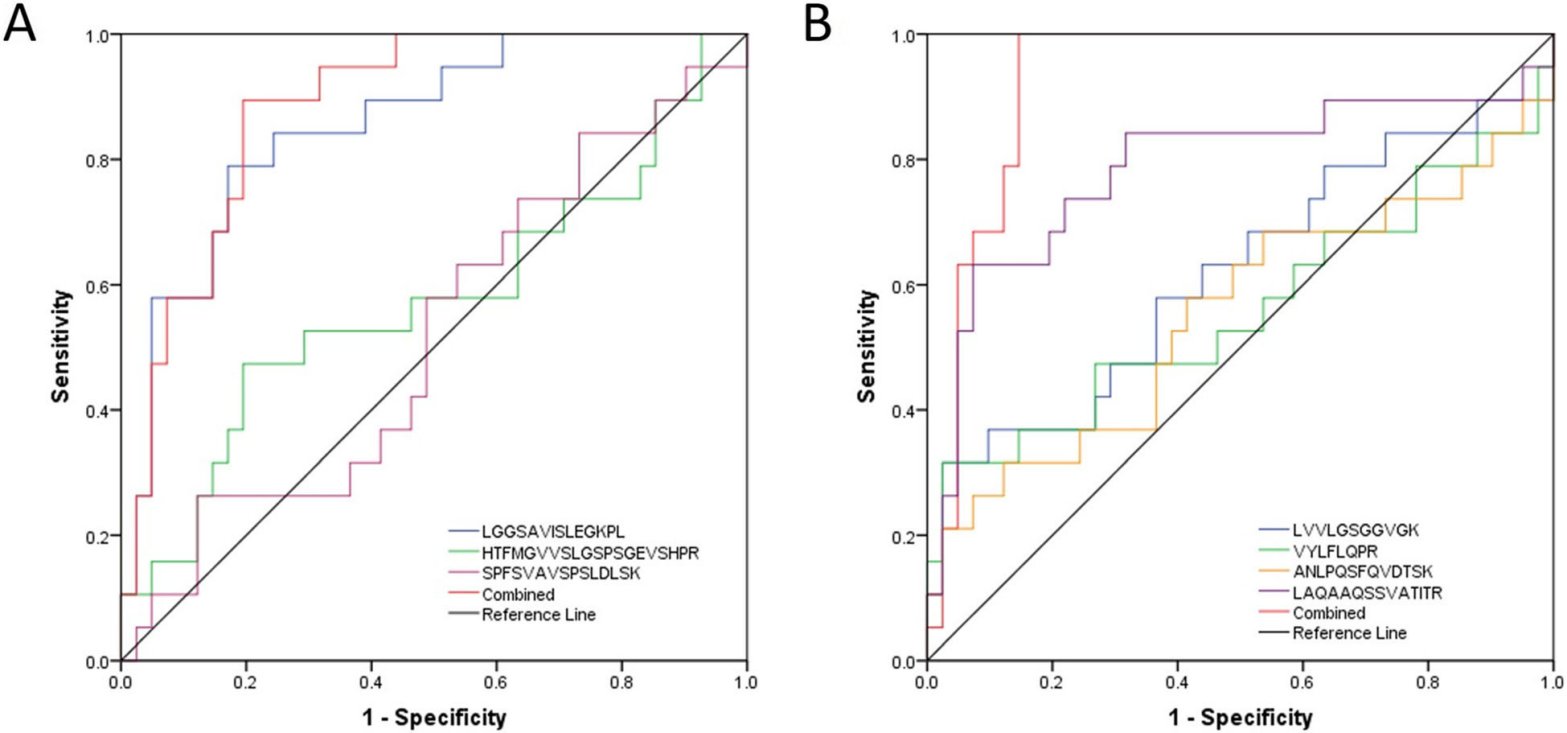
Receiver operator curve analysis of 19 cases and 41 controls by multivariate logistic regression of individual or combined peptides. Model 1 (**a**) includes peptides LGGSAVISLEGKPL (CFL1), HTFMGVVSLGSPSGEVSHPR (AHSG), and SPFSVAVSPSLDLSK (FLNA). Model 2 (**b**) includes peptides LVVLGSGGVGK (RAP1A), VYLFLQPR (ITGA2B), ANLPQSFQVDTSK (FLNA), and LAQAAQSSVATITR (TLN1)

Twenty-four proteins among the 56 found in the discovery phase were selected for verification in an independent cohort using LC-MRM. Of the 24 candidates, 41 proteotypic peptides were selected for MRM analysis (Table 3). The peptides were quantified in the verification cohort of 19 breast cancer patients at stage I (T1N0M0) and in 41 controls including serum samples obtained from women with inflammatory diseases and benign breast pathology findings (Table 1, Validation Set). All 41 peptides were shown to be more abundant in sera from breast cancer patients, and 11 were statistically significant (*p* value ≤ 0.01) (Supplementary Excel Table, sheet: “Wilcoxon test”). We then performed logistic regression analysis to determine if marker combinations showed superior sensitivity and specificity compared to single markers. Model 1 was built using three proteins previously identified as potential biomarkers of breast cancer in blood and/or tissue: Cofilin 1 (CFL1, LGGSAVISLEGKPL), Alpha-2-HS-glycoprotein (AHSG, HTFMGVVSLGSPSGEVSHPR), and Filamin A (FLNA, SPFSVAVSPSLDLSK). CFL1 was the only one that showed a significant difference between IDC cases and controls (Fig. 3). However, CFL1 was also differentially abundant in our discovery set in lung cancer and therefore not specific to IDC. Using all three proteins, model 1 yielded a sensitivity of 89.47% and a specificity of 80.49%. For these predictors, PPV and NPV was 68% and 95.3%, respectively, with an AUC of 0.88 (Table 4, Fig. 4a). Model 2 included four candidate predictors specific for breast cancer in this study: Ras-related protein Rap-1A (RAP1A, LVVLGSGGVGK), Integrin alpha-IIb (ITGA2B, VYLFLQPR), FLNA (ANLPQSFQVDTSK), and Talin-1 (TLN1, LAQAAQSSVATITR). Using univariate analysis, only TLN1 was significantly different between cases and controls. However, the combination of the four markers in model 2 outperformed the single marker, achieving a sensitivity of 100% and specificity of 85.37%. PPV and NPV were 76% and 100% (*p* < 0.05), respectively, with an AUC of 0.93 (Table 4, Fig. 4b). In both models, the combined markers outperformed single markers (Supplementary Table S5). Two peptides from FLNA (ANLPQSFQVDTSK, SPFSVAVSPSLDLSK) were identified separately as predictors for the two models, but the correlation between their levels as measured by LC-MRM was low (Supplementary Fig. 3).

**Table 3:** Peptide candidates selected for the MRM assay

Gene	Peptide sequence	Precursor ***m/z***	Precursor charge
FERMT3	VFVGEEDPEAESVTLR	888.93	2
	VVLAGGVAPALFR	635.38	2
ACTG1	GYSFTTTAER	566.77	2
ACTG1/POTEF	AGFAGDDAPR	488.73	2
	AVFPSIVGRPR	599.86	2
ACTN (ACTN1/ACTN4)	VGWEQLLTTIAR	693.89	2
	LASDLLEWIR	608.34	2
GP1BB	LSLTDPLVAER	607.34	2
RAP1 (RAP1A/RAP1B)	LVVLGSGGVGK	493.31	2
	SKINVNEIFYDLVR	570.65	3
	SALTVQFVQGIFVEK	833.46	2
TUBA	EIIDLVLDR	543.31	2
	LISQIVSSITASLR	744.44	2
TUBA	VGINYQPPTVVPGGDLAK	913.00	2
TUBB1	GASALQLER	472.76	2
	EVDQQLLSVQTR	708.38	2
TUBB (TUBB1/TUBB3/TUBB6)	FPGQLNADLR	565.80	2
ITGB3	SKVELEVR	480.28	2
PFN1	STGGAPTFNVTVTK	690.36	2
	TFVNITPAEVGVLVGK	822.47	2
CFL1	LGGSAVISLEGKPL	670.89	2
BMP1	LNGSITSPGWPK	628.84	2
LTF	DGAGDVAFIR	510.76	2
ITGA2B	VAIVVGAPR	441.28	2
	VYLFLQPR	518.30	2
THBS1	SITLFVQEDR	604.32	2
	GFLLLASLR	495.31	2
FLNA; FLN1	ANLPQSFQVDTSK	717.86	2
	YGGQPVPNFPSK	645.83	2
	SPFSVAVSPSLDLSK	767.41	2
MYH9	ALELDSNLYR	597.31	2
HPSE	FLILLGSPK	494.32	2
	TDFLIFDPK	548.29	2
AHSG	HTFMGVVSLGSPSGEVSHPR	699.68	3
TLN1	LAQAAQSSVATITR	708.89	2
	ILAQATSDLVNAIK	728.92	2
	GLAGAVSELLR	543.32	2
TLN2	VMVTNVTSLLK	610.85	2
	SIAAATSALVK	516.31	2
MST1	VVGGHPGNSPWTVSLR	831.94	2
LIMS2	VIEGDVVSALNK	622.35	2

Peptides were selected using the following criteria: highest number of identified peptides and intensities from discovery dataset, presence of at least 3 high abundant *− b* and/or − *y* product ions, highest ESP scores and scored transitions in the PeptideAtlas, no Met residues, and + 2 or + 3 charge state precursor ions. The protein isoforms are described in parenthesis

**Table 4:** Summary of logistic regression values for biomarkers predicting group status

	AUC (95% CI)	Criterion	Sensitivity	Specificity
Model 1				
LGGSAVISLEGKPL	0.86 (0.76–0.96)	> 0.25	78.95	82.93
HTFMGVVSLGSPSGEVSHPR	0.57 (0.40–0.74)	> 0.34	47.37	80.49
SPFSVAVSPSLDLSK	0.52 (0.36–0.68)	> 0.32	26.32	87.80
Combined	0.88 (0.80–0.97)	> 0.25	89.47	80.49
Model 2				
LVVLGSGGVGK	0.62 (0.45–0.78)	> 0.47	31.58	97.56
VYLFLQPR	0.56 (0.38–0.74)	> 0.39	31.58	97.56
ANLPQSFQVDTSK	0.55 (0.38–0.73)	> 0.35	31.58	87.80
LAQAAQSSVATITR	0.79 (0.64–0.93)	> 0.37	63.16	92.68
Combined	0.93 (0.86–1.00)	> 0.19	100	85.37

Model 1 was run on 41 potential peptide biomarkers with *p* < 0.05. Significant predictors from model 1 were tested using model 2. Logistic regression was used to determine the sensitivity, specificity, and area-under-curve (AUC) of single markers and combined panels of peptide biomarkers, after bootstrapping 1000 samples with 95% confidence intervals for each specified cutoff value of the criterion. *CI* confidence interval

## Discussion

The development of a minimally-invasive molecular assay using blood-borne/circulating biomarkers to support mammography screening is highly desirable to increase PPV for early-stage breast cancer detection while limiting the number of unnecessary biopsy of benign conditions that mammography cannot currently discriminate from frank early malignancy.

In this study, we uncovered 56 candidate protein biomarkers of IDC breast cancer (and/or protein fragments) in serum. When we compared the 56 candidate proteins with proteins increased in patient sera across the other cancer cohorts, 32 were altered in IDC but not in prostate, ovarian, colon or lung cancer, or melanoma or in their matched benign/inflammatory control cohorts.

The strategy of protein enrichment by HNs showed to be effective for the identification of rare/low abundance serum proteins. Proteins known to circulate in blood (plasma/serum) at low concentrations such as CCL14 (~ 5 ng/mL [22]), BMP1 (~ 50 ng/mL [23]), and heparanase (~ 100 pg/mL [24]) were identified.

Many of the proteins upregulated in the serum of breast cancer patients had never been previously observed in serum using LC-MS/MS, such as paired-like homeodomain transcription factor 2 isoform b (PITX2), AKT3 kinase isoform 2 (AKT3), tissue inhibitor of metalloproteinase 3 precursor (TIMP3) [25, 26], and Tropomyosin alpha-4 chain (TMP4). TIMP3 and TMP4 are relevant to breast cancer biology and both were reported to be more abundant in tumor interstitial fluid collected from triple negative breast cancer patients compared to normal interstitial fluid [27]. Queries using ProteomicsDB (https://www.ProteomicsDB.org) and The Human Protein Atlas (2020-07-27) showed that none of the identified proteins were exclusive to breast cancer cell lines or breast tissue (data not shown). However, even if our discovery study across multiple cancers suggested that the proteins are more abundant in blood of individuals with breast cancer, as compared to cancers in other organs, we did not expect them to be specific to breast cancer tumors. Indeed, our study shows a high degree of specificity of those markers as circulating proteins in breast cancer patients.

According to the NCI-funded Breast Cancer Surveillance Consortium (HHSN261201100031C, downloaded on 2019/02/06), sensitivity and specificity calculated over more than two million screening mammography examinations are 85.6% and 90.5%, respectively.

Our model 1 discriminated cases and controls with a sensitivity of 89.5% and a specificity of 80.5%, outperforming the specificity of the single markers reported previously. In particular, plasma levels of the Filamin-A (FLNA) 280 kDa variant predict metastatic breast cancer with 96.7% sensitivity but only 67.8% specificity [28]. In model 2, the individual markers achieved very high specificity (> 90%), but low sensitivity. However, the combination of the four markers dramatically increased the sensitivity (100%), while still maintaining adequate specificity (85%).

Our study supports the results of previous breast cancer biomarker studies and highlights a significant presence in the serum of IDC patients of proteins derived from the extracellular matrix and associated to cell proliferation, migration, adhesion, and metastasis.

Using the “Analysis of protein sets” function of Proteomics DB (Supplementary Fig. 4), revealed four proteins included in model 2 (ITGA2B, FLNA, RAP1A, and TLN1) are widely expressed across different type of tissues. However, ITGA2B shows to be particularly enriched in platelet, non-small cell lung cancer cells (ProteomicsDB), and basophils (Human Protein Atlas). The presence of circulating ITGA2B may indicate an ongoing process of tumor cell-induced platelet aggregation [29].

We observed increased levels of AHSG in serum of breast cancer patients. In a previous study, anti-Alpha-2-HS-glycoprotein (AHSG) antibodies were detected in 33 of 36 patients with breast cancer (91.7%) [30]. The two studies support the hypothesis that changes at the level of expression, but possibly also to location, PTMs or structure, occur to AHSG during breast cancer, such as to induce the production of autoantibodies.

Cofilin 1 (CFL1) is an intracellular actin-modulating protein associated with EGF-stimulated chemotaxis [31] and invadopodia localization in breast cancer cell invasion [32]. Although overexpression of CFL1 in breast cancer tissue has been associated with poor prognosis and survival [33], to our knowledge serum levels of CFL1 were never assessed. Talin 1 (TLN1) is a cytoskeletal protein that functions in extravasation and breast cancer cell migration [34, 35]. High levels of both TLN1 and CFL1 are reported in the secretome of breast cancer cell lines (including, metastatic, triple-negative, MCF7 ER-positive, and triple-negative) [36], confirming their release into the ECM. In addition to increased TLN1 abundance, we observed an increased abundance of integrin and RAS oncogene family members in IDC patients, suggesting that an ongoing dynamic remodeling of the cytoskeleton involving integrins and active regulation of adhesion molecules by TLN1 is detectable in serum due to an increase level of these proteins.

Integrin alpha-IIb (ITG2AB) plays a role in breast cancer metastasis through its role in matrix cross-linking processes [37]. ITG2AB polymorphisms are associated with increased breast cancer risk [38]. RAP1A, a member of the RAS oncogene family, regulates signaling associated with proliferation, adhesion, and migration mediated by beta1 integrin levels [34, 35, 39].

FLNA was found to be a predictor in both models, but with two different peptides. Both cover a region common to isoforms 1 and 2 and are found in the secreted form of FLNA (280 kDa) N-terminal side to the calpain cleavage site. Levels of both peptides showed relatively low correlation with each other (Pearson correlation coefficient = 0.51) (Supplementary Fig. 3). In targeted mass spectrometry experiments, this phenomenon is attributed to variability in digestion efficiency, the presence of missed cleavages, protein modifications, different isoforms, or differential enzymatic degradation. However, SPFSVAVSPSLDLSK may be modified by phosphoserine (pS968) [36, 40] while no modifications likely occur in ANLPQSFQVDTSK. Moreover, ANLPQSFQVDTSK levels correlate positively with a third peptide YGGQPVPNFPSK (Pearson correlation coefficient = 0.96), which is likely unmodified and was not a significant factor in any of our diagnostic models (Supplementary Fig. 3). Interestingly, SPFSVAVSPSLDLSK and ANLPQSFQVDTSK contribute to different models: SPFSVAVSPSLDLSK participates in the first model, which comprises proteins specific to both breast cancer sera and homeostatic response pathways, while ANLPQSFQVDTSK participates in the second, which includes only breast specific proteins and favors a strong motility component.

We postulate that the breast cancer protein signature described here reflects the changing ECM and stromal composition of the breast cancer tissue microenvironment during early tumorigenic processes. As observed in rodent models, breast tissue is characterized by high stromal content and is particularly rich in fibrillar collagens and matricellular proteins [25, 28] involved in the activation of adhesion signals [37, 41] [42] and enhancing invasion and metastasis [43]. ECM remodeling occurs during the early stages of IDC and results in leakage of proteins and protein fragments into the circulation. Shen et al. observed that breast tumors shed cancer-specific peptides and products of proteolytic activity into circulation [44]. Our data reinforces the hypothesis that this shedding of ECM components into the circulation even at the earliest stages of malignancy can be used to design a specific and sensitive biomarker panel to improve detection of breast cancer.

Although this study uses an innovative nanoparticle-based protein capture technique that focuses on the LMW portion of the proteome to identify candidate protein/protein fragments and peptides as serum biomarkers of breast cancer, as well as a unique collection of multi-tumor serum sets and matched benign conditions, a separate discovery and verification set including semiquantitative MRM-based verification of selected candidates, there are some limitations to our study.

The use of spectral counting in our discovery cohort may have limited the accuracy of the analysis of semi-quantitative label-free data. Many other approaches for spectral counting and ion intensity normalization [45, 46] have been explored. Although our candidates were independently verified using an LC-MRM method, we recognize that use of semi-quantitative label-free data employed in the discovery phase may be a limitation of the study. Other alternative nanoparticle-based sample processing techniques provide broad proteomic coverage of blood proteins [47]. Unlike our HN sample enrichment method, these approaches do not specifically enrich the extra-vascular content contained in the LMW low abundant blood proteome.

Relevant to FLNA peptides, we emphasize that the HN-CB/VSA serum pre-processing is aimed at analysis of low molecular weight proteins. Therefore, we cannot rule out that circulating fragments belonging to FLN1 or other high molecular weight proteins might be enriched by HN and further digested to tryptic peptides after elution.

Moreover, while our discovery and verification sets were carefully constructed to maximize chances of identification of specific and sensitive markers for early breast cancer detection, the study sets were inherently unbalanced and do not reflect the frequency of occult/non-detected breast cancer in the general population. However, the number and type of samples chosen for this study was consistent with the objectives of discovery, qualification and verification of potential breast cancer biomarkers. Additional validation studies will require not only a larger population of individuals comprised of different BI-RADS scores and molecular subtypes [48], but also a thorough and stringent validation of our MRM methods, following the Tier for validation suggested by the National Institutes of Health, the National Cancer Institute (CPTAC - Clinical Proteomic Tumor Analysis Consortium), and National Heart, Lung, and Blood Institute (Proteomics Centers) [49].

In conclusion, these findings would require much more intensive validation in blinded, independent study sets in order to judge the potential for clinical impact. Our results, taken together, justify such further validation.

## Conclusion

Our objective was to use a series of innovative sample processing and proteomic approaches coupled to a unique sample study set discover and validate a panel of candidate serum proteins that could potentially be used to detect early-stage breast cancer as an adjunct with mammography. We developed a semi-quantitative MRM assay that employs a simple method of protein enrichment, representing a robust foundation suitable for future validation studies. We also discovered a panel of proteins, which were validated in an independent cohort from largely early-stage (T1a) breast cancers vs serum taken from women with benign/inflammatory conditions as a control set, with a sensitivity and specificity profile that could have clinical impact when combined with mammography. Further studies on larger cohorts of individuals who were subjected to mammography will be required to clarify if our proposed protein panel can complement the diagnostic performance of mammography.

## Supplementary information

**Additional file 1: Table S1.** Cancer cohort characteristics. **Table S2.** Peptides used as internal standards in this study. **Table S3.** E.Coli peptides used to evaluate instrumental variance in the LC-MRM validation assay. **Table S4.** Candidate peptide transitions employed for the LC-MRM validation assay. **Table S5.** Logistic regression analysis and validation of candidate peptide markers. **Figure S1.** Frequency distribution and histograms of molecular weight (A) and abundance (B) for unique plasma proteins identified across cases and controls. Enrichment of low abundance, low molecular weight proteins using nanoparticle capture is shown. **Figure S2.** Overlap of pathways identified by functional enrichment analysis across control and IDC cases (stages I-III). Enriched pathways were filtered for *p* < 0.05 and relevance in > 50% of samples. **Figure S3.** Correlation analysis of Filamin A peptides AUC in models 1 and 2. Pearson’s correlation coefficients are shown for each correlation pair. Analysis excludes outlier cases 22 and 44. Starred correlations: *p* < 0.001. **Figure S4.** Expression heatmap generated using the ProteomicsDB online analytical tool (https://www.proteomicsdb.org/).

## Data Availability

The data used in the present study are available from the corresponding author upon reasonable request.

## Abbreviations

SDF-1β: Stromal cell-derived factor 1β
IGFBP7: Insulin-like growth factor binding protein 7
IGF1: Insulin-like growth factor 1
MRM: Multiple reaction monitoring
PPV: Positive predictive value
NPV: Negative predictive value
ECM: Extracellular matrix
