# Supplementary material for: Shotgun proteomics coupled to nanoparticle-based biomarker enrichment reveals a novel panel of extracellular matrix proteins as candidate serum protein biomarkers for early-stage breast cancer detection
Source: Breast Cancer Res. 2020 Dec 2;22:135. doi: 10.1186/s13058-020-01373-9 (PMC7709252; doi:10.1186/s13058-020-01373-9)

**Supplementary Tables**

**Supplementary Table S1. Cancer cohort characteristics**

| Cancer type | Information | Cases | Controls |
| --- | --- | --- | --- |
| Lung | Sex: male/female | 14/6 | 13/7 |
|  | Age (years): mean±SD | 64.5±8.3 | 63.75±6.64 |
|  | Stage: IA-B/IIA-B/IIIA-B | 8/6/6 |  |
|  | Benign conditions (none/hamartoma/thoracotomy/carcinoid) |  | 14/2/2/2 |
|  | Smoke habits: YES/IN THE PAST/NEVER | 9/7/4 | 6/9/5 |
| Colon | Gender: male/female | 11/9 | 10/10 |
|  | Age (years): mean±SD | 59.55±8.77 | 57.9±12.24 |
|  | Stage (IA-B/IIA-B/IIIA-B/NA) | 7/5/7/1 |  |
|  | Benign conditions (negative colonoscopy/inflammatory bowel disease/adenoma) |  | 5/5/10 |
| Prostate | Gender: male/female | 20/0 | 20/0 |
|  | Age (years): mean±SD | 64.1±6.52 | 65.8±5.90 |
|  | Gleason Score (5/6/7) | 2/10/8 |  |
|  | PSA (ng/mL) | pre*7.48±2.89  post** 0.025±0.049 | 6.01±2.25 |
| Melanoma | Gender: male/female | 15/5 | 11/9 |
|  | Age | 62.9±20.50 | 51.1±13.71 |
|  | Breslow thickness | 2.67±1.25 |  |
|  | Nevus classification (nc, ng, reed, blue) |  | 14/4/1/1 |
| Ovarian | Gender: male/female | 0/20 | 0/20 |
|  | Age(years): mean±SD | 55.0±10.57 | 55±10.86 |
|  | Stage (IA-B/IIA-B/IIIB-C/NA) | 3/1/16/0 |  |
|  | Menopause (NO/YES) | 9/11 | 11/9 |
|  | Benign conditions (cervicitis/condiloma/cystadenoma/leiomyoma/ovarian cysts/fibrothecoma/polyps/teratoma/NA) |  | 6/1/1/3/4/1/2/1/1 |

^*^Pre-prostatectomy/**Post-prostatectomy

**Supplementary Table S2.** Peptides used as internal standards in this study

| Protein Gene | Protein Description | Peptide Sequence | Precursor *m/z* | Precursor Charge |
| --- | --- | --- | --- | --- |
| Lyz | Lysozyme | GYSLGNWVCAAK | 663.31 | 2 |
|  |  | FESNFNTQATNR | 714.82 | 2 |

**Supplementary Table S3.** *E.Coli* peptides used to evaluate instrumental variance in the LC-MRM validation assay

| Peptide Sequence | Precursor *m/z* | Precursor Charge |
| --- | --- | --- |
| GVNANHIIR | 497.3 | 2 |
| LNIDQNPGTAPK | 634.3 | 2 |
| ALEGDAEWEAK | 609.8 | 2 |
| YIVALDQGTTSSR | 705.9 | 2 |
| IAAANVPAFVSGK | 622.9 | 2 |
| LAATIAQLPDQIGAK | 755.4 | 2 |
| STLTPVVISNMDEIK | 823.9 | 2 |
| EMLPVLEAVAK | 600.3 | 2 |

**Supplementary Table S4.** Candidate peptide transitions employed for the LC-MRM validation assay

| Gene | Peptide Sequence | Precursor *m/z* | Precursor Charge | Transition list |
| --- | --- | --- | --- | --- |
| FERMT3 | VFVGEEDPEAESVTLR | 888.93 | 2 | 1116.6(*y*10*^+^*),1001.5(*y*9*^+^*),775.4(*y*7*^+^*),575.4(*y*5*^+^*),765.9(*y*10^+2^) |
|  | VVLAGGVAPALFR | 635.38 | 2 | 958.5(*y*10*^+^*),887.5(*y*9*^+^*),674.4(*y*6*^+^*),603.4(*y*5*^+^*),536.3(*y*11^+2^) |
| ACTG1 | GYSFTTTAER | 566.77 | 2 | 912.4(*y*8*^+^*),825.4(*y*7*^+^*),577.3(*y*5*^+^*),476.2(*y*4*^+^*) |
|  | AGFAGDDAPR | 488.73 | 2 | 701.3(*y*7*^+^*),630.3(*y*6*^+^*),458.2(*y*4*^+^*),424.7(*y*8*^+2^*) |
|  | AVFPSIVGRPR | 599.86 | 2 | 514.8(*y*9*^+2^*),441.3(*y*8*^+2^*),450.3(A9*^+2^*) |
| ACTN1 | VGWEQLLTTIAR | 693.89 | 2 | 787.5(*y*7*^+^*),674.4(*y*6*^+^*),561.3(*y*9*^+^*) |
|  | LASDLLEWIR | 608.34 | 2 | 1031.6(*y*8*^+^*),716.4(*y*5*^+^*),603.3(*y*4*^+^*) |
| GP1BB | LSLTDPLVAER | 607.34 | 2 | 900.5(*y*8*^+^*),799.4(*y*7*^+^*),684.4(*y*6*^+^*),507.3(*y*9*^+2^*) |
| RAP1A | LVVLGSGGVGK | 493.31 | 2 | 773.5(*y*9*^+^*),674.4(*y*8*^+^*),561.3(*y*7*^+^*),387.2(*y*9*^+2^*) |
|  | SKINVNEIFYDLVR | 570.65 | 3 | 812.4 (*y*6*^+^*),665.4 (*y*5*^+^*),406.7 (*y*6*^+2^*),785.4 (*b*7*^+^*) |
|  | SALTVQFVQGIFVEK | 833.46 | 2 | 1194.7 (*y*10*^+^*),1066.6 (*y*9*^+^*),919.5(*y*8*^+^*),522.3(*y*4*^+^*) |
| TUBA1C; TUBA6 | EIIDLVLDR | 543.31 | 2 | 730.4(*y*6*^+^*),615.4(*y*5*^+^*),502.3(*y*4*^+^*),403.2(*y*3*^+^*) |
|  | LISQIVSSITASLR | 744.44 | 2 | 933.5(*y*9*^+^*),834.5(*y*8*^+^*),375.2(*y*3*^+^*),442.3(*b*4*^+^*) |
| TUBA3E | VGINYQPPTVVPGGDLAK | 912.9 | 2 | 1150.6 (*y*12*^+^*),657.4(*y*7*^+^*),675.3(*b*6*^+^*), 395.2(c7*^+2^*),494.3(c9*^+2^*) |
| TUBB1 | GASALQLER | 472.76 | 2 | 658.4(*y*5*^+^*),545.3(*y*4*^+^*),408.7(*y*7*^+2^*) |
|  | EVDQQLLSVQTR | 708.38 | 2 | 400.2(*b*5*^+^*),703.4(*y*6*^+^*),590.3(*y*5*^+^*), 404.2(*y*3*^+^*) |
| TUBB6 | FPGQLNADLR | 565.8 | 2 | 886.5(*y*8*^+^*),701.4(*y*6*^+^*),588.3(*y*5*^+^*),843.4(*b*8*^+^*) |
| ITGB3 | SKVELEVR | 480.28 | 2 | 744.4(*y*6*^+^*),516.3(*y*4*^+^*),416.3(*a*4*^+^*) |
| PFN1 | STGGAPTFNVTVTK | 690.36 | 2 | 1006.6(*y*9*^+^*),503.8(*y*9*^+^*),374.2(*b*5*^+^*) |
|  | TFVNITPAEVGVLVGK | 822.47 | 2 | 1069.6(*y*11*^+^*),968.6(*y*10*^+^*),698.4(*y*14*^+2^*),575.3(*b*5*^+^*) |
| CFL1 | LGGSAVISLEGKPL | 670.89 | 2 | 856.5(*y*8*^+^*),414.3(*y*4*^+^*),485.3(*b*6*^+2^*),1112.6(*b*12*^+^*) |
| BMP1 | LNGSITSPGWPK | 628.84 | 2 | 1029.5(*y*10*^+^*),885.5(*y*8*^+^*),772.4(*b*7*^+^*),671.4(*b*6*^+^*) |
| LTF | DGAGDVAFIR | 510.76 | 2 | 777.4(*y*7*^+^*),605.4(*y*5*^+^*),506.3(*y*4*^+^*),435.3(*y*3*^+^*),416.1(*b*5*^+^*) |
| ITGA2B | VAIVVGAPR | 441.28 | 2 | 499.3(*y*5*^+^*),400.2(*y*4*^+^*),383.3(*b*4*^+^*),482.3(*b*5*^+^*) |
|  | VYLFLQPR | 518.3 | 2 | 773.5(*y*6*^+^*),660.4(*y*5*^+^*),513.3(*y*4*^+^*),400.2(*y*3*^+^*),376.2(*b*3*^+^*) |
| THBS1 | SITLFVQEDR | 604.32 | 2 | 793.4(*y*6*^+^*),646.3(*y*5*^+^*),504.3(*y*8*^+2^*),397.2(*y*6*^+2^*) |
|  | GFLLLASLR | 495.31 | 2 | 672.4(*y*6*^+^*),559.4(*y*5*^+^*),446.3(*y*4*^+^*),375.2(*y*3*^+^*),544.3( *b*5*^+^*) |
| FLNA; FLN1 | ANLPQSFQVDTSK | 717.86 | 2 | 911.4(*y*8*^+^*),450.2(*y*4*^+^*),625.3(*y*11*^+2^*),568.8(*y*10*^+2^*) |
|  | YGGQPVPNFPSK | 645.83 | 2 | 885.5(*y*8*^+^*),689.4(*y*6*^+^*),478.3(*y*4*^+^*),406.2(*b*4*^+^*) |
|  | SPFSVAVSPSLDLSK | 767.41 | 2 | 1016.6(*y*10*^+^*),945.5(*y*9*^+^*),846.5(*y*8*^+^*),759.4(*y*7*^+^*) |
| MYH9 | ALELDSNLYR | 597.31 | 2 | 1,009.5(*y*8*^+^*),880.5(*y*7*^+^*),767.4(*y*6*^+^*),652.3(*y*5*^+^*) |
| HPSE | FLILLGSPK | 494.32 | 2 | 727.5(*y*7*^+^*),614.4(*y*6*^+^*),501.3(*y*5*^+^*),388.2(*y*4*^+^*) |
|  | TDFLIFDPK | 548.29 | 2 | 879.5(*y*7*^+^*),732.4(*y*6*^+^*),619.3(*y*5*^+^*) |
| AHSG | HTFMGVVSLGSPSGEVSHPR | 699.68 | 3 | 965.5(*y*9*^+^*),868.4(*y*8*^+^*),724.4(*y*6*^+^*) |
| TLN1 | LAQAAQSSVATITR | 708.89 | 2 | 1033.6(*y*10*^+^*),834.5(*y*8*^+^*),616.8(*y*12*^+2^*),455.3(*b*5*^+^*),607.8(*a*13*^+2^*) |
|  | ILAQATSDLVNAIK | 728.92 | 2 | 1031.6(*y*10*^+^*),960.5(*y*9*^+^*),445.3(*y*4*^+^*),615.8(*y*12*^+2^*) |
|  | GLAGAVSELLR | 543.32 | 2 | 844.5(*y*8*^+^*),716.4(*y*6*^+^*),617.4(*y*5*^+^*),370.2(*b*5*^+^*),469.3(*b*6*^+^*) |
| TLN2 | VMVTNVTSLLK | 610.85 | 2 | 974.6(*y*9*^+^*),875.5(*y*8*^+^*),561.4(*y*5*^+^*),460.3(*y*4*^+^*) |
|  | SIAAATSALVK | 516.31 | 2 | 831.5(*y*9*^+^*),760.5(*y*8*^+^*),689.4(*y*7*^+^*),618.4(*y*6*^+^*) |
| MST1 | VVGGHPGNSPWTVSLR | 831.94 | 2 | 1213.6(*y*11*^+^*),450.2(*b*5*^+^*),422.3(*a*5*^+^*) |
| LIMS2 | VIEGDVVSALNK | 622.35 | 2 | 1031.5(*y*10*^+^*),902.5(*y*9*^+^*),631.4(*y*6*^+^*),532.3(*y*5*^+^*) |

**Supplementary Table S5. Logistic regression analysis and validation of candidate peptide markers**

|  | Observed Coefficient | Bootstrapped Standard Error | *P*- value | 95% CI  Lower Limit | 95% CI Upper Limit |
| --- | --- | --- | --- | --- | --- |
| **Model 1** | |  |  |  |  |
| LGGSAVISLEGKPL (CFL1) | 7.54E^-7^ | 1.00E^-5^ | 0.021 | 2.36E^-7^ | 7.00E^-6^ |
| HTFMGVVSLGSPSGEVSHPR (AHSG) | -7.15E^-9^ | 4.68E^-7^ | 0.788 | -3.38E^-7^ | 3.65E^-8^ |
| SPFSVAVSPSLDLSK (FLNA) | 6.74E^-9^ | 6.07E^-9^ | 0.717 | -7.74E^-8^ | 9.89E^-8^ |
| Model Sensitivity | 89.5% |  |  |  |  |
| Model Specificity | 80.5% |  |  |  |  |
| PPV | 68.0% |  |  |  |  |
| NPV | 94.3% |  |  |  |  |
| AUC | 0.88 |  |  |  |  |
| **Model 2** | |  |  |  |  |
| LVVLGSGGVGK (RAP1A) | -5.96E^-9^ | 0.000015 | 0.887 | -1.53E^-7^ | 1.22E^-7^ |
| VYLFLQPR (ITGA2B) | 9.04E^-9^ | 0.000008 | 0.434 | -1.18E^-7^ | 7.69E^-8^ |
| ANLPQSFQVDTSK (FLNA) | -1.80E^-7^ | 0.000009 | 0.005 | -9.07E^-7^ | -7.26E^-8^ |
| LAQAAQSSVATITR (TLN1) | 4.83E^-7^ | 0.000101 | <0.001 | 2.68E^-7^ | 3.00E^-6^ |
| Model Sensitivity | 100% |  |  |  |  |
| Model Specificity | 85.37 |  |  |  |  |
| PPV | 76.0% |  |  |  |  |
| NPV | 100% |  |  |  |  |
| AUC | 0.93 |  |  |  |  |

Marker panels predicted from logistic regression models 1 and 2, using 41 peptide candidates with p <0.05 from the validation dataset. Bootstrapped standard errors were obtained using 1000 samples with a range of 95% CI limits. PPV, positive predictive value; NPV, negative predictive value; AUC, area under the curve.

**SUPPLEMENTARY FIGURES**

**Supplementary Figure 1. Frequency distribution and histograms of molecular weight (A) and abundance (B) for unique plasma proteins identified across cases and controls.** Enrichment of low abundance, low molecular weight proteins using nanoparticle capture is shown.


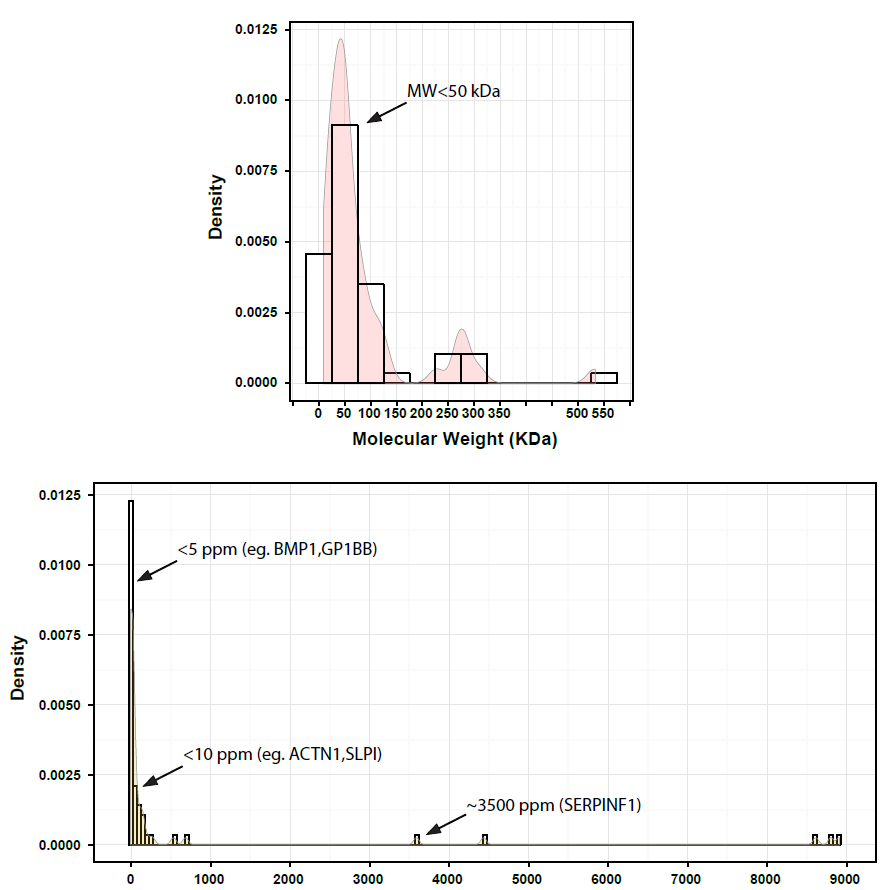


**Supplementary Figure 2: Overlap of pathways identified by functional enrichment analysis across control and IDC cases (stages I-III).** Enriched pathways were filtered for *p*<0.05 and relevance in >50% of samples.


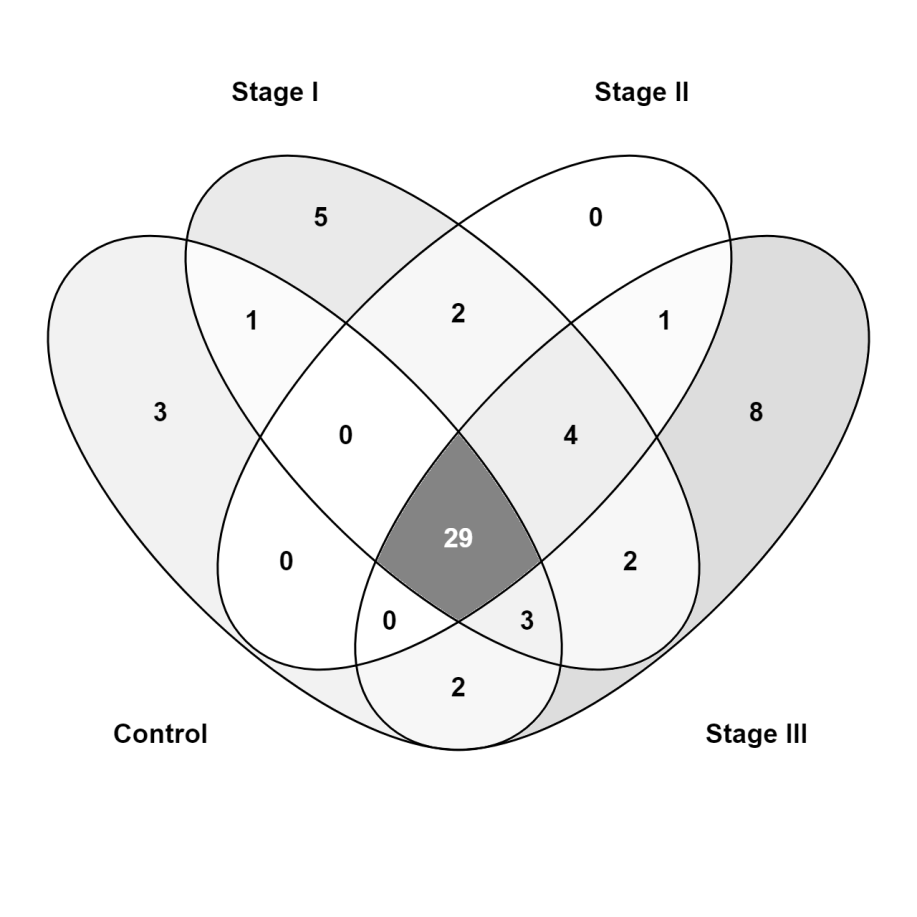


**Supplementary Figure 3: Correlation analysis of Filamin A peptides AUC in models 1 and 2.** Pearson’s correlation coefficients are shown for each correlation pair. Analysis excludes outlier cases 22 and 44. Starred correlations: p<0.001.


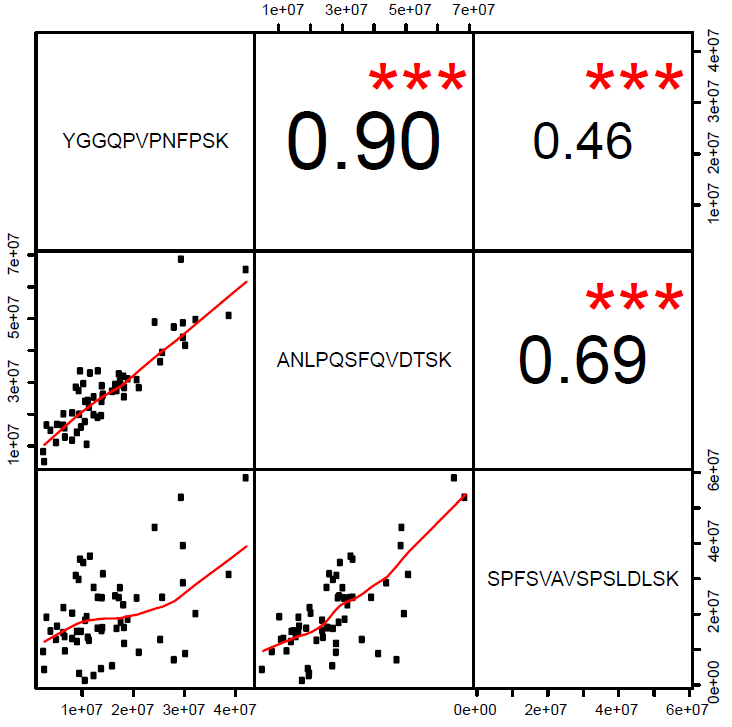


**Supplementary Figure 4. Expression heatmap generated using the ProteomicsDB online analytical tool (**[**https://www.proteomicsdb.org/**](https://www.proteomicsdb.org/)**).** A two-way hierarchical clustering analysis representing protein expression across human tissues for ITGA2B, FLNA, RAP1A and TLN1 (proteins included in model 2). Protein Accession numbers are the following ITGA2B (B7Z8B0);ITGA2B (P08514);ITGA2B (P08514-2) ;ITGA2B (P08514-3);

FLNA (H7C2E7); RAP1A (P62834);FLNA (H0Y5F3);FLNA (F8WE98);FLNA (E9PHF0);FLNA (A6NDY9);

FLNA (Q5HY54); FLNA (P21333); FLNA (P21333-2); FLNA (H0Y5C6);TLN1 (Q5TCU6); TLN1 (Q9Y490).


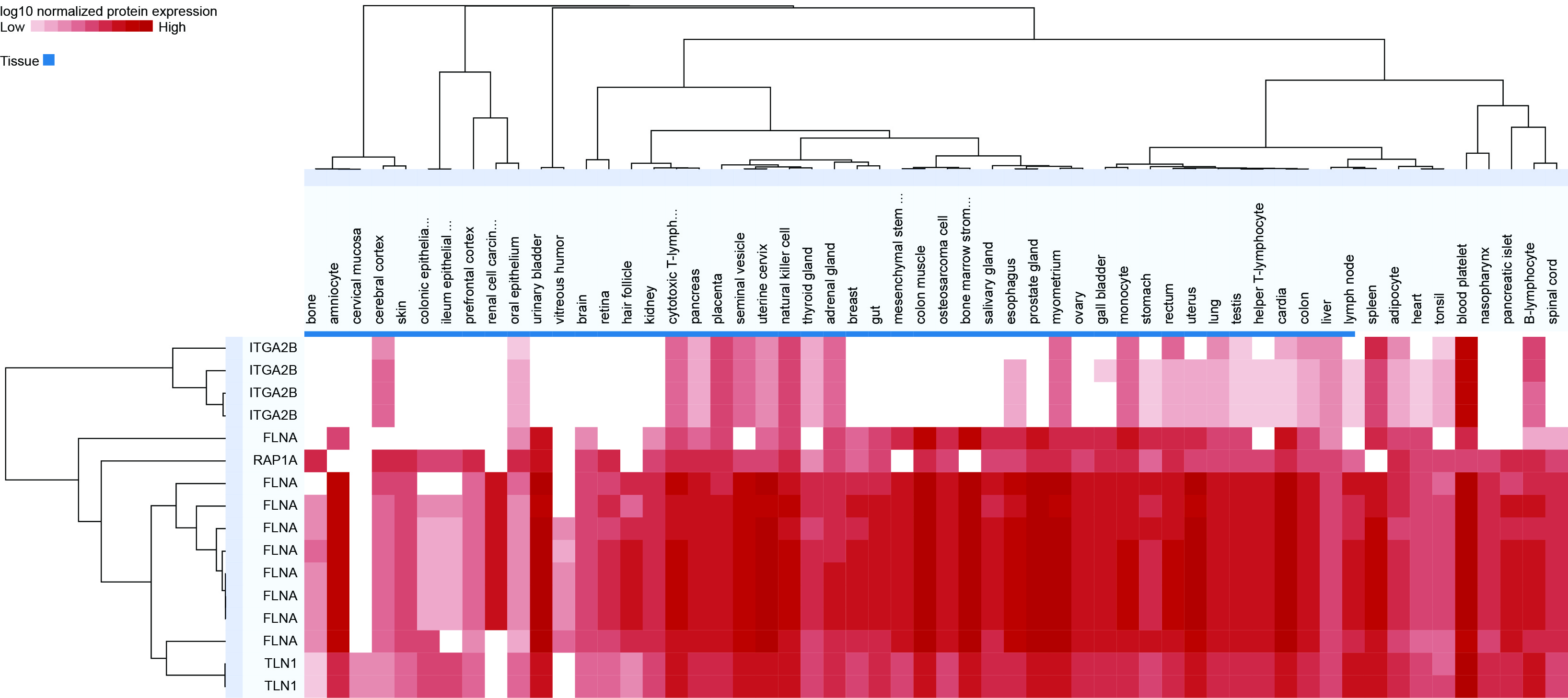

Supplement: Supplementary file 1 — Additional file 1: Table S1. Cancer cohort characteristics. Table S2. Peptides used as internal standards in this study. Table S3. E.Coli peptides used to evaluate instrumental variance in the LC-MRM validation assay. Table S4. Candidate peptide transitions employed for the LC-MRM validation assay. Table S5. Logistic regression analysis and validation of candidate peptide markers. Figure S1. Frequency distribution and histograms of molecular weight (A) and abundance (B) for unique plasma proteins identified across cases and controls. Enrichment of low abundance, low molecular weight proteins using nanoparticle capture is shown. Figure S2. Overlap of pathways identified by functional enrichment analysis across control and IDC cases (stages I-III). Enriched pathways were filtered for p < 0.05 and relevance in > 50% of samples. Figure S3. Correlation analysis of Filamin A peptides AUC in models 1 and 2. Pearson’s correlation coefficients are shown for each correlation pair. Analysis excludes outlier cases 22 and 44. Starred correlations: p < 0.001. Figure S4. Expression heatmap generated using the ProteomicsDB online analytical tool (https://www.proteomicsdb.org/). [file 13058_2020_1373_MOESM1_ESM.docx]
